# Sabotage, feeding and collusion after bariatric surgery. And the winner is . . .? A psychodynamic and systemic perspective on sabotage and feeding after bariatric surgery by means of a case series analysis

**DOI:** 10.1177/13634593251319928

**Published:** 2025-02-27

**Authors:** Friedrich Stiefel, Laurent Michaud, Céline Bourquin-Sachse, Sophia Quirke-McFarlane, Jane Ogden

**Affiliations:** University Lausanne and University Hospital Lausanne, Switzerland; University Lausanne and University Hospital Lausanne, Switzerland; University Lausanne and University Hospital Lausanne, Switzerland; University of Surrey, UK; University of Surrey, UK

**Keywords:** bariatric surgery, feeding, sabotage, collusion

## Abstract

In the context of bariatric surgery, negative social support has recently been conceptualized in terms of sabotage, feeding behaviour and collusion undermining a person’s effort to lose or maintain weight. While sabotage and feeding behaviour are thought to be motivated consciously, collusion is understood as a bond, by which protagonists are tied together sharing an unresolved and unconscious psychological issue such as dependency, domination and submission, and so on. Drawing upon a systemic and psychodynamic understanding, we analysed interviews with patients (*n* = 10) who had undergone bariatric surgery and their partners (*n* = 10) focusing on support. We selected interviews (*n* = 4 + 4), illustrative of sabotage, feeding, collusion and co-evolution based on the comprehensiveness of information, their emblematic quality and suitability to delineate these phenomena. Our analysis confirms that negative social support can be considered as an attempt to reestablish a level of homeostasis within the couple. However, rather than being intentional, we consider that sabotage and feeding behaviour are better conceptualised as consequences of collusive relationships.

## Introduction

The role of social support, especially of partners (Butterfield and Lewis, 2002; Ma, 2015), in weight loss, weight regain and weight maintenance of persons living with obesity has long been recognized (Thomas et al., 2008). Whilst most research focuses on the positive impact of social support in obesity management (Whale et al., 2014), there is also a problematic side such as the lack of social support (Kayman et al., 1990) and attitudes and behaviours of significant others, which can have negative consequences (Scambler et al., 1981).

In a recent analysis of existing theories and research, Ogden and Quirke-McFarlane (2023) argued that “social support” can also be detrimental to a person’s weight management attempts and proposed a “new model of negative social support” referring to (i) sabotage (active and intentional undermining of another person’s weight goals), (ii) feeding behaviour (explicit overfeeding of someone who is not hungry or wishing not to eat) and (iii) collusion (to avoid conflict). These three types of negative social support are conceptualized by the authors within the context of relationships considered as systems with mechanisms of homeostasis and have been supported by qualitative analysis of patient experiences of sabotage by their partner and quantitative assessments of feeder motivations and behaviour (Ogden et al., 2020; Ogden et al., 2022; Quirke-McFarlane & Ogden, 2024). To date, less is known about the collusion component of this model.

Our study aimed to take the next step to test Ogden and Quirke-McFarlane’s (2023) model of negative social support by means of an analysis of a case series of interviews with patients living with obesity, who have undergone bariatric surgery, and their partners.

To inform the readers, we will first briefly describe the three types of negative social support proposed by these authors. We then present the methodology used for the case series approach. Exemplary quotes together with our analysis, which argue for and discuss our perspective are inserted in each case vignette, which illustrate situations of sabotage, feeding, collusion (without an effect on eating behaviour) and co-evolution.

## Sabotage: Cui bono?

“Sabotage” denotes attitudes and behaviours, which undermine the efforts of the person living with obesity to adopt a healthier lifestyle and to lose or maintain weight (Befort et al., 2008; Hardcastle and Hagger, 2011; Hindle and Carpenter, 2011; Metzgar et al., 2015). Sabotage occurs for example as discouraging healthy eating, putting barriers to attending support groups or physical activity, or indirectly as lowering mood through criticism and hurtful comments. Reasons motivating sabotage, thought to operate actively or inadvertently and consciously or unconsciously (Kluever Romo and Dailey, 2014; Mauro et al., 2008; Rogerson et al., 2016), are envy of the person losing weight or guilt of not losing weight (Moore and Cooper, 2016); suspicion of infidelity when partners loses weight or fear that they might wish to separate (Mauro et al., 2008); shift of the balance from an equal relationship with both partners being overweight to an asymmetrical relationship, which contains unpredictability and uncertainty (Kluever Romo and Dailey, 2014); or tensions due to dietary changes (Paisley et al., 2008). In their model, Ogden and Quirke-McFarlane (2023) consider that sabotage is intentional.

## Feeder behaviour: It’s not always about food

The term “feeding behaviour” originally denoted sexual gratification through having another person eat, who is not hungry (Bestard, 2008), but Ogden and colleagues propose to use it for “explicit overfeeding of someone who is not hungry or does not wish to eat.” To date, research exploring feeder behaviour has identified motivations relating to waste avoidance, affection, manners or status, with feeding being reciprocal or unidirectional (Ogden et al., 2020; 2022; Ogden & McFarlane, in press).

## Collusion: A short history of the concept with a working definition

Ogden and Quirke-McFarlane (2023) consider collusion as a “more benign way” of negative social support, a “core part of communication” between individuals and a means to “maintain conversation and avoid conflict”; compared to sabotage and feeder behaviour, collusion seems less intentional and often “reflect kindness, friendship and support.” Collusion has been defined in many ways, depending on disciplines (e.g. social psychology, psychoanalysis, or system psychodynamic theory) and settings (e.g. medical, psychiatric or management setting) (Stiefel et al., 2023, 2024). Conceptually, the spectrum of collusion reaches from conscious intentional attitudes, words and actions to unconscious relational dynamics.

While descriptions of collusions date back to early psychoanalytic writings, the concept appeared under many different names in the psychiatric literature such as impasse, bastion, enactment or blind spots (Stiefel et al., 2023). The term “collusion” was introduced by Henry Dicks in 1967 (Dicks, 2014) to describe couples, who are linked by an unconscious bond, which explains that each partner’s (pathological) behavior can only be understood by considering their relational dynamics. Jürg Willi, a couples therapist, popularized collusion among the public with his book “Couples in Collusion” in 1984 (Willi et al., 1982) and provided a classification of collusion depending on the dynamics and developmental difficulties involved (e.g. oral collusions over the issue of passivity and activity, anal-sadistic collusions over domination and submission, oedipal collusions over sexual identity and narcissistic collusions over self-esteem).

To empirically investigate collusions, as defined by Dicks and Willi, who anchored collusion in a systemic and psychodynamic perspective, we developed a working definition, based on two literature reviews (Stiefel et al., 2023, 2024). Thereafter, we tested this definition by means of a clinical case series of collusion-centered supervisions of oncology clinicians, which allowed us to delineate collusion from other transference-countertransference experiences (Deliyanidis et al., 2023).

This working definition is based on the following characteristics: collusion is (i) a specific relational dynamic between two or more persons, (ii) who share an unresolved and unconscious issue, (iii) by which they are interlocked in a defensive manoeuvre. The issue at stake is (iv) avoided on an intrapsychic level (v) by means of externalization (e.g. projection, projective identification or acting), which (vi) entertains a vicious circle (“pouring oil on fire”). The unresolved issue may pertain to control, intimacy, loss, harm, dependency, domination, boundaries, and so on (Stiefel et al., 2017, 2023). While colluders experience distress and may recognize their difficulties, they do not recognize, handle or work through the issue at stake and underlying dynamics, and instead avoid it by acting in the relationship. Collusion can be either symmetrical (colluders adopt the same stance towards the issue, e.g. power, with both trying to dominate in the relationship) or complementary (e.g. one dominates and the other submits).

## Material and methods

To investigate sabotage, feeder behaviour and collusion we carried out a case series analysis of patients who had undergone bariatric surgery to explore how these components of negative support played out in their relationships. Using the definition of sabotage and feeding behaviour provided by Ogden and Quirke-McFarlane (2023), we relied on the definition of collusion provided by Dicks and Willi and operationalized as a working definition by Stiefel et al. (2023).

## Participants and interviewer

Patients were recruited by means of an online advertisement posted via social media and email invitations to participants in an ongoing study. Eligible patients were aged > 18 years old, able to converse in English, had undergone bariatric surgery more than 1 year ago, were in partnership at least 1 year prior to surgery, and had a partner who has not undergone bariatric surgery. Thirty patients took part in the interview; participants were then asked to invite their partners to participate in an interview. Ten partners agreed to take part. This resulted in 10 matched dyads. The interviews were conducted by SQMF separately with each partner. The interviewer, a female, White British, not living with obesity and not having had bariatric surgery, health psychologist, was at the time of the study in training. Given the absence from personal experiences with obesity and her youth, certain participants may have felt less inclined to open up. We feel, however, that the interviewer mitigated against these potential issues by using open questions, active listening, being interest and non-judgmental regarding issues of living with obesity and by her warmth and relational qualities. The study was approved by the local ethics committee and participants provided informed consent.

## Methodology

The interviews were conducted with the aim to investigate negative social support occurring in couples with one or both partners living with obesity. The study was based on a semi-structured interview guide, which was developed by one of the authors (JO), who is a Professor in Health Psychology with a specific interest in obesity, and the interviewer, and based on their prior research experiences (see Table 1, submitted as supplementary material). The interview focused on the following main topics: social support received from the partner after bariatric surgery over the journey prior and after surgery, and perception by the partner of support provided. The overall aim of the interview was first explained, and the questions were completed, where necessary, with additional information or examples, facilitating participants to understand their meaning. Questions were framed in an easily understandable English. Table 1 lists the questions and prompts of the interview guide and provides an idea of the procedure and different areas, the interview aimed to investigate.

## Data analysis

The first author (FS), a senior psychiatric liaison clinician, trained as a psychodynamic-oriented psychiatrist and psychotherapist read the verbatim-transcribed interviews and selected interviews (*n* = 4 + 4) (purposive sampling), based on the comprehensiveness of information and their illustrative quality and suitability to delineate sabotage, feeding, and collusion. From this process the notion of co-evolution was also identified, and a case selected.

FS summarized and commented on the interviews in a written document, which was submitted, together with the original interviews, to the co-authors, who were senior (JO) and junior (SQMF) researchers with a focus on obesity and social support, a senior liaison psychiatrist (LM), who supervises a bariatric surgery clinic, and a social scientist (CB), who has extensive experience with qualitative studies in the medical and psychiatric setting. CB, LM and FS have already conducted empirical research on collusion. The summaries were compared to the full-length interviews by a psychology student, who verified that they correspond to the transcriptions. The co-authors validated, invalidated, or completed the commentaries to the summaries; consensus was reached in a second round.

## Epistemological position

Epistemologically, qualitative analysis takes an inductive perspective with the researcher deriving themes and patterns from the data. In contrast, quantitative analysis tends to take a more hypothetico-deductive approach through testing a priori hypotheses. Given existing definitions of our key constructs and the use of an existing model it was not the aim of the present study to take a purely inductive approach as this would deny our a priori position. Further, given the use of dyadic interviews illustrating the opportunity for novel insights, the present study did not aim to utilise a purely hypothesis testing approach. In line with this, the present study therefore utilised an iterative position between both inductive and deductive perspectives. Furthermore, the study drew upon a combined systemic and psychodynamic approach as suggested by Willi (1984). A systemic perspective is based on general system theory, which understands not only the functions of the different elements of a system, but their interrelations; social coexistence is thus viewed as a complex and integrated whole, which is greater than the sum of its parts (Minuchin, 1988; Sameroff et al., 1983). This systemic perspective also conceptualises couples as organized by elements, which have identifiable attributes and functions, and interrelations which can be understood (De Vries and Stiefel, 2018). The psychodynamic perspective is based on Freud’s work, object relation theory derived from Klein’s and Winnicott’s work, and self-psychology derived from Sullivan’s interpersonal work (Ellman, 2020; Lewin, 2005). This perspective relies on assumptions such as the existence of an unconscious, which influences our thoughts, emotions and behaviours, defence mechanisms which mediate between an individual’s inner and outer world or the relevance of past relational difficulties, which can be re-enacted in relationships (De Vries and Stiefel, 2018). When we use the notion of “systemic” or “psychodynamic” perspective, we do not consider them as an etiological model to explain eating disorders or obesity but as perspectives, which can help to understand how individuals and couples relate to the disease, and how they interact together, with others and the environment. Accordingly, we developed a prior notion of sabotage, feeder behaviour and collusion from existing literature and ‘tested’ these through a psychodynamic and systemic analysis of qualitative interviews.

## Findings

Our analysis presents four case studies illustrating the key concepts of sabotage, feeder behaviour and collusion and how these play out within couples. Furthermore, the analysis identified an interactional dynamic of co-evolution. The four case studies are presented as summaries with an analysis and exemplar quotes, as well as verbatims illustrating some of the pronouncements on which the analysis was based and allowing to gain a more complete picture of the participants, for example through the style, choice of words, expression used, etc.

### Case study 1: Sabotage: Rocky days—The woman who is not the person she used to be and the man who “lost” his wife after bariatric surgery

Heather, who underwent bariatric surgery 3 years ago, observes that she changed during the journey of bariatric surgery (“I’m not the person I used to be”). She reports that she is now attentive to eating healthy, doesn’t go out that often anymore and stopped drinking alcohol and overeating.

H1: I feel happy. I’m a lot happier. I feel healthier. And obviously a lot smaller. H2: I mean, if he goes out now and brings something home, I won’t have it. You know, I am really strict with myself. You know, don’t get me wrong, I do have occasional treats, but I prefer not to have stuff like that.

This is confirmed by her husband, Emmett, himself living with obesity, who now goes out on his own and eats and drinks too much, considering that his wife is obsessive about her weight despite that she has lost already a lot. He states that the changes are hard from a social perspective and complains that his wife sometimes “snaps at him”, that she is subjected to mood swings and makes him question if he did something wrong. He feels that the time after surgery hasn’t been “a bed of roses”, that life changed a lot and that they experience “rocky days”.

E1: Sometimes when we go out as a couple before, before she had the operation, we used to go out and have a drink together. She still comes out and has a drink maybe, but it’s very, very hit and miss now when she does come out; I still go out and have a beer but I’d rather [name of wife] comes out with me sometimes. E2: I think we’ve got used to that she eats this way now and I’ve got used to it. Sometimes she’s a little bit obsessive about her weight. Even though she’s lost a hell of a lot. She’s kind of worries still. E3: I always ask her if she’s ok, and then she gets fed up with me sometimes and snaps at me, yes!

The relational dynamics of the couple have been modified by surgery. It seems the couple had a stable relationship in the past, both living with obesity and regulating their emotions by oral compensations. One can make the hypothesis, that they were bond by an oral collusion, which has been perturbated by Heather’s changing lifestyle and behaviour (her mood swings may also be related to the impossibility to resort to old mechanism of oral compensation).

Furthermore, Heather considers that her husband did not support her enough prior and after bariatric surgery; she states that he could have encouraged her more, shown more curiosity regarding her medical appointments, complimented her when she put on new clothes after having lost weight or enhanced her confidence in herself. Moreover, Heather observes that he was at times jealous and reports an event where he accused her of flirting with an old friend, which was according to her not at all the case.

H3: I think just sort of saying, not really giving me confidence. And saying, you know, you look good, or, you know, like I said before, you know about wearing different, different things. H4: We were just sort of like talking just outside of the hall. And he got really funny with me, you know, where have you been? Oh, and all this sort of thing. I said, oh, you know, just talking. And he said, well he really fancies you, you know? You know, because you’ve lost weight, blah, blah, blah. And I have never seen that before, never seen that before, never ever.

Emmett observes that his wife lacks confidence (due to excess skin and reduction of breast size after bariatric surgery) and needs a lot of reassurance. He also complains and repeatedly reports during the interviews that they had no sex since surgery, which he considers unfair, regretting that this topic is pushed under the carpet; he thinks that his wife should see a doctor to overcome these difficulties. He repeatedly states that he always supports his wife’s decisions but that supporting her decisions (e.g., bariatric surgery) does not imply that he always agrees. He admits, however, that he also nags at her.

E4: She’s not very body confident where she’s lost some weight, she has some saggy skin and things like that. Her breast size has reduced quite a lot and things like that. . . . She needs reassurance quite a lot. She really does. I suppose it comes down to sex. We haven’t had sex properly since she had the operation. I don’t like to talk about this, but I’ll talk about it. [Name of the wife] has gone to see a doctor and I think she’s currently seeing a doctor, but I’ve told her she’s got to snap out of where she is, because it’s not fair. It’s just not fair on me. E5: I’ve been supportive? I’ve always been there for her. I haven’t always agreed with everything. I’m not gonna say I have.

One could assume, that the couple is linked by a shared unresolved issue over self-esteem, which was covered in the past by the collusive relationship and the associated oral compensations. Heather now needs reassurance and Emmett feels jealous. Both partners express complaints pointing to low self-esteem. Moreover, the fact that Emmett accepts that issues are pushed under the carpet and often silently disagrees is a supplementary clue. Heather’s snapping and refusal of sex may be interpreted as (passive) aggressiveness; the same holds true for Emmett’s indifference, nagging and lack of support. Passive-aggressive reaction out of frustration may be interpreted as retaliations toward the other, who does not serve to stabilise self-esteem.

Heather observes that her husband continuously buys unhealthy food even though she tells him that she doesn’t want to eat it, that he is frustrated about the social restrictions which followed surgery and annoyed by her acquired capacity to renounce to fatty food. However, Heather considers that Emmett does not bring home the “fat stuff” on purpose. His remarks concerning her new social and eating habits (“you never used to be like this”) are hurting her.

H5: I’d say you, you know, if he went out to the pub, he’d say, well you know, I’ll bring a takeaway home. And I‘d say, no, I don’t want a takeaway. But that didn’t always work. H6: And I think he found that very frustrating. The social aspect of it. H7: And, you know [he says], you never used to be like this. Or, you know, you used to do that or . . .

*Emmett admits that he is annoyed but affirms that he still loves her, “she’s my wife”.*

E6: And then if we go out for a meal, sometimes with the food portion, I end up, she ends up wasting most of it, which is kind of, I suppose, I am a little bit annoyed, really.

The couple thus seems to be interlocked by an unconscious, interpersonal defensive dynamic with externalization and avoidance of own difficulties on the intrapsychic level. The unconscious manifests itself by a lack of recognition regarding the couple’s dynamic, with each partner perceiving the deficits of the other, but without acknowledging own difficulties. They externalize their frustration either by “snapping” and “nagging” at each other, by an attitude of unnegotiable refusal (a kind of “sexual” sabotage by Heather) or by disrespect for the modified lifestyle and eating behaviour (sabotage by Emmett). One could say that the compensated symmetrical oral collusion has turned into a decompensated symetrical oral collusion with frustration and aggressiveness. The lack of self-confidence, which has been laid bare by surgery, remains unaddressed on an intrapsychic level and the couple is engaged in a collusive vicious circle, mutually attacking the other’s self-confidence. The mutual attacks contribute to lower self-esteem, which leads to the entertainment of the collusive dynamic (“pouring oil on fire”). What speaks thus in favour of a collusive relationship is the couple’s impossibility to evolve with the new situation; on the contrary, the relationship has worsened, and each partner contributes to this by means of interpersonal defences (such as projection and projective identification and acting out) and externalization of unaddressed and unresolved intrapsychic issues. Unable to provide what his wife externalizes as demands (support for her lack of self-confidence, which was not resolved by surgery), the husband—suffering from the same unresolved issue—wishes to delegate this key demand to a third party, a clinician. Collusive frustration, oral aggressiveness and sabotage are the consequence.

### Case study 2: Feeder behaviour: “It’s a weird change in the balance of the relationship” —The woman who doesn’t need to lean on her husband anymore and the man who lost his mission

Olivia, who underwent surgery 3 years ago, observes that “a weird change in the balance of the relationship” with her husband has occurred after surgery. Indeed, her husband, who was her personal trainer in the past, used to encourage her to exercise up to 4 times a week and during weekends, prepared her healthy food and was always positive and supportive, while she was a negative person. Meanwhile he has changed. Once “more complimentary” when she was “bigger,” Billy has gone “the complete opposite way”: he now “took a step back,” ceases to be supportive and stops complimenting her; moreover, he has gained weight and doesn’t care about exercises anymore. It is now up to her to stimulate him to do more for his health, without success.

O1: So, my husband was an ex personal trainer. So, I used to quite a lot of personal training classes with him. O2: So, he was very supportive, and certainly in trying to help me lose weight, and to encourage that he always knew that I was unhappy. O3: Him being an ex-PT, he’s gone the complete opposite way. He can’t stand exercise now. He doesn’t do it. O4: So my goals, if you like, have changed and I’ve morphed into him, and he’s morphed into me a little. So yeah, so it’s a weird change in the balance of the relationship, if that makes sense.

This is confirmed by Billy, who states that before he was into exercise but now suffers from knee issues. He confirms that his wife has changed and is more active physically (she now enjoys sport independent of its potential to decrease weight) and socially. He also conveys that before surgery, his wife did not lose weight despite all their efforts, considering that meanwhile she lost too much weight and that he can’t understand why she still wants to lose more weight.

B1: I lived, breathed, and slept fitness, much to her dismay. You know, literally nothing else mattered in my life. And now it’s gone the other way. B2: I have gained weight and really couldn’t care less, and she’s at the gym. B3: She keeps saying, oh, you know, you should come out with me. And I’m like, No. I’m not gonna for a run. I’m too old now, my legs are all shot to pieces. B4: But sometimes she still feels that she’s lost not enough.

The relational dynamics of the couple seems to evolve around activity and passivity or who is stimulating whom, which can also be understood—according to Willi’s typology of collusion—as an oral problematic (one is nurturing, and the other is nurtured).

Olivia reports that she needed surgery because of severe physical health issues, not only for aesthetic reasons. Since then, she feels better and enjoys the new possibilities life offers her. Billy, on the other hand, reports that nothing mattered more in his life than fitness (he was not only trainer, but he also participated in fitness shows) but that he now suffers from mental health issues, on which he no further elaborates, and considers not to be able to exercise anymore.

The shared unresolved issue revolves around activity and passivity (or progression and regression), in an alternating dynamic within the couple. Only one of the partners can be active, progressive and adopt a healthy behaviour, the other must remain passive, regressive and indifferent to health. Indeed prior to surgery, all of Olivia’s efforts to lose weight remained unsuccessful despite Billy’s efforts. Now Billy remains inactive and adopts unhealthy eating behaviours despite Olivia’s efforts to stimulate him. A collusive reversal of roles has taken place.

Since surgery, according to Olivia, Billy has gained weight (he always fluctuated during his life), and she considers his inactivity as being related to laziness (“he is a bit lazy”) or a lack of time (“he doesn’t have the time”). He has ceased to compliment her, telling her “You look absolutely fine” and “you don’t need to go back to the gym”. He also worries that she doesn’t eat enough, encourages her to finish the plates and asks her why she doesn’t eat more. On the morning of her surgery, when he drove her to the hospital and she was starving and not allowed to eat, he stopped by a McDonald’s to order breakfast for him and told her to “hold it while they’re reversing out of the space.” Olivia then reports that she has body dysmorphophobia issues. Finally, now that Olivia has gained weight again during the COVID-19 pandemic, she observes that Billy “jumps on the bandwagon” and is quite supportive again. Billy (as evidenced by his limited responses to the interviewer’s proses and prompts) reports information but does not further explore them searching for underlying motivations and refers only once to a psychological dimension when he observes that Olivia’s continuous wish to lose weight, which he doesn’t share, may be related to her “personality.”

The unconscious, interpersonal defensive dynamic, with externalization and avoidance of own difficulties on the intrapsychic level appears at different moments of the interviews. Both partners perceive the psychological problems of the other, but they are not psychologized and interpreted as “laziness,” “not enough time” or attributed to “the personality.” Both admit own psychological problems but fail to elaborate on them, just labelling them as disorders. The swings in the relational dynamic, with Billy becoming again quite supportive after Olivia has gained weight remains unquestioned. Both engage in actions and not reflections; their activity and passivity are neither addressed on the interpersonal nor on the intrapsychic level. Attitudes of activity and passivity dominate the interpersonal relationship. Actions, also circulate around food, and seem to replace verbal communication. Olivia grows from a dependant follower into an autonomous individual, while Billy regresses from a supportive saviour into a dependant and unsupportive feeder, and switches back again in a more active role as soon as Olivia regains weight. These swings are an important element in favour of collusion: they illustrate the relational bond, which entertains stagnation and hampers evolution. Feeding behaviour can be understood as a desperate attempt by Billy to re-establish the old pre-bariatric surgery situation. Unlike the above discussed situation of sabotage, in which the husband somehow “lost” his wife, Billy’s situation seems more dramatic: he also lost his mission (as a personal trainer) during this journey. The partners recognize the role reversal but not its underlying mechanisms. The reversal of the relationship continues that “oil is poured on fire”; a complementary collusion persists with weight loss being compensated with weight gain, resulting in a zero-sum game. In contrast to the previous couple, the relationship thus seems to be more stable and less under pressure.

### Case study 3: Collusion—The more he insists, the less he gets, the more she wants, the less she receives

Maggie reports that prior to bariatric surgery, her husband was not much of a support. Both were big eaters and used to go to all-you-can-eat-buffets, trying to get their money’s worth. She was always the instigator to eat, while he first refused to then quickly agree to follow her. According to Maggie, this fact—she is taking the lead and he is following her—has been an essential characteristic of their couple, since she considers that her husband is insecure about anything (e.g. regarding his capacities to drive a car, his job performance or their relationship coloured by the anxiety of being left by her).

M1: But we both had, we both did have really big appetites. And we would go to the eat as much as you can buffets. M2: So, I was always the instigator. M3: He’s insecure about everything. You name it. He’s insecure about it.

Jacob considers that the surgery provides Maggie with confidence; she no longer must go out “covered up” with “many layers even in 40 degrees heat.” These changes had positive effects on their relationship, except when they are outside the house and she gets hungry, and the available food is inadequate for her: in such situations some friction may emerge. Moreover, since surgery, their communication improved, and he perceives his wife as less “reclused” (before, “she wouldn’t tell me things”).

J1: And I think this is a life changer, I really do. It’s nice to see, it’s a pleasure to see. **J2:** You know as regards the relationship, it’s the only time it gets hard, so I think when we are trying to discuss meals . . . and then they haven’t got the stuff there that she needs. . . . You know, so it causes a little bit of friction there. J3: We talk now, whereas before she was a bit of, I wouldn’t say recluse, it’s probably a bit too hard of a word, but she wouldn’t tell me things. But now we’re totally open, she discusses things, even sort of personal things.

The relational dynamics of the couple can be viewed as a symmetrical oral collusion with one in a progressive position and the other globally insecure and in a regressive dependent position.

According to Maggie, prior to surgery, Jacob was not that supportive. Despite the fact that he was always attracted to women living with overweight (his ex-wife, girlfriends), he now supports her - except exercise-wise, he isn’t up to that - in her attempts to lose weight (e.g., gives up food, which is not good for her; states that she shouldn’t buy it for him; accepts that she leaves food on the plate confirming that the portion was too big). On the other side, some of his comments hurt Maggie; for example, when he points out her extra skin, underlines that she has lost her curves or complains that she renounces dressing up. Surprisingly, since surgery, her husband seems less preoccupied by the fear that she might leave him. Moreover, Maggie resents that he enjoys buying her clothes and underwear, “wants her to show her body off”, something she never did, and wishes she dressed like a twenty-year-old (she is 54). She ends the interview by stating that it was always difficult for her to let people down, because of her insecurities, that she is not relaxed about food and that she has the impression she only lives “half a life”; however, she doesn’t dare to tell her husband about such issues she struggles with.

M4: Before surgery? I wouldn’t say there was much support before surgery. M5: His first wife was a big lady. I was really a big lady. And a couple of girlfriends that I think he had in between, they were big as well. And he does like curvaceous women. M6: When I had a bit of a problem with the crisp and cheesy crackers sort of thing, and I was buying them for him. . . . And then I said, Yeah, but you like them, he said, Yeah, but they’re not good for me either. So he said, don’t buy them. M7: It’s mainly the excess skin, when he comments about it, and he says, you know, oh, your bingo wings sort of thing. And I don’t think he realises how much it hurts. M8: I feel sort of a 54-year-old woman dressing as a 54-year-old woman, not 54 dressing as a 20 because that would be awful. M9: I don’t feel as if I can relax around food and my life. . . . Because I still got my issues with food. . . . So I feel at the moment, I’m only living half a life.

Jacob more than extensively elaborates how he supports Maggie with her weight loss, how he adapts his own meals, accompanies her to medical appointments, reads information on weight loss or searches for adequate food when they are outside. He also admits that he sometimes still orders a drink for her in the restaurant forgetting that he shouldn’t do this and that he might sometimes create some uneasiness, when he keeps hampering her to dress “like a woman”. For him, the main element of change in their relationship, a 100% change, is his joy to see her being able to just go into a shop and buy clothes: no more need for to “cover up”: he gladly remembers her smile when she first walked into a shop and could buy anything. Jacob ends the interview, after repeating that surgery was a life changer, with “Now I’ve got to worry about the weight (his own weight) and everything.”

J4: I mean, it’s a hard one because I think, because she was so set in her way, in the way she dressed and the way she went out and that I kept hammering to dress more like a woman or whatever, something that caused a little bit of uneasiness. J5: It’s worked brilliantly. Absolutely fantastic, it honestly has. Because as I say, she’s got more confidence now, just to go out, whatever.

Here, the shared unresolved issue surfaces: self-confidence. Prior to surgery, this issue was covered by oral compensation in a symmetrical collusion, which has been turned after surgery into a collusion over gender and sexual identity. Now, it’s Jacob who occupies the progressive position: he strives to “feminize” his wife, possibly not only to help her to break her “cover up,” but also to experience himself this break-up by collusive proxy (vicarious pleasure). However, he admits that it is also now up to him to take care of his weight. Maggie resists, since she feels uncomfortable in the attributed role of a sexually attractive young women, which ignores reality and herself as a person. She has never experienced such desires and feels thus estranged and hurt by Jacob’s remarks and insisting demands.

Maggie complains about Jacob’s unsupportive way of being with her. However, Jacob considers that with his insistence on her dressing up, he is “just trying to build her confidence back up” and wants her now to be happy (“we’re note the youngest people anymore”).

J6: You’ve got your figure now. What, why would you not want to do something with that? Show it! Before you used to moan about that I’m not wearing that, I’m not wearing that. Now, I said you’ve got it, you can do it. To start with, it was, I think was getting her confidence up.

The unconscious, interpersonal defensive dynamic, with externalization and avoidance of own difficulties on the intrapsychic level is especially observable in Jacob, who seems to enjoy experiencing (by proxy) the new situation, somehow attempting to compensate for past renunciations. These attempts, however, produce the contrary, with Maggie refusing to follow his desires, feeling alienated from her husband. Surgery changed a silent symmetrical oral collusion into a noisy complementary collusion with Jacob sexualizing Maggie, who refuses herself to him. This constellation qualifies, according to Willi, as a phallic-oedipal collusion; this type of collusion denotes couples in which the gender roles are exaggerated and play a major role in the relationship. The couple’s shared intrapsychic problems with self-confidence, also over gender roles and sexuality, remain unaddressed and are externalized (Maggie desiring an unconditionally supportive husband and Jacob desiring a wife who takes pride in her new post-bariatric surgery appearance by dressing up). While the collusive dynamic is mainly situated on a psychological level and not on the level of eating behaviours as in the first two situations, one observes of a vicious cycle entertained by both protagonists who “poor oil on fire” (the more Jacob insists, the less Maggie will be willing to comply and the more Maggie demands from Jacob what he cannot provide, the less she will receive). Jacob’s perception of new possibilities regarding his wife and the couple invades their relationship, which collusively stagnates, unable to evolve. There is no new future, even if they are “not yet old” as Jacob has put it. The new configuration—like all collusions—reflects a relationship in which each partner is not encountered as he or she is but maintained in a role (which he or she might accept or refuse), which corresponds to own psychological needs.

### Case study 4: Co-evolution: “We are pretty good at helping each other”—social support in a non-collusive relationship

Owen, who underwent bariatric surgery 3 ½ years ago, describes himself as having been often in denial regarding his eating behaviours but benefiting from his wife’s honest “voice of reason,” which enabled him to stop brushing eating and weight issues under the rug. He feels that the couple works well together and explains how his wife played an important role by providing practical help (e.g. meal plans, medication, banning sweets from the home, reduction of portions, etc.) but also mental health support (e.g. finding treats, little things he could look forward to, enabling him to become independent from food, health education, etc.). The only unhelpful element he can think of, was that his wife first hided herself when eating, while he considered that he had to learn to be able to face this situation, even or because his food envy will never be solved.

O1: So, it’s you know, it was the support, was there to sort of try and stop and curb bad eating tendencies and try to push forward better eating and better sort of lifestyle. I would say being the voice of reason to a degree because a lot of time I found myself in denial. O2: Um, I’d say the biggest sort of support is working together . . . O3: My wife at first, the first couple of months didn’t eat dinner around me. And she would hide her food, like the first weeks after hospital. And I wondered why I hadn’t eaten with her yet sort of thing. And I mentioned it. . . . and she’s hiding from me because I was so hypersensitive with smell, smelling foods was that, but yeah, that’s the only thing is food envy, but that won’t ever go away.

Owen’s views are confirmed by Alice, who states that the couple is “pretty good at keeping each other honest”. She considers that the relationship improved after surgery, since in the past she saw Owen helplessly struggling with weight issues, a situation which somehow created a “rift” between them, facing such different experiences. This was also the reasons she was able to understand that Owen felt at times better understood by looking at videos of patients with weight problems witnessing their experiences than by her. Besides the practical help, Alice tried to encourage Owen by taking weekly pictures and videos, which she collated to show him how he improved and increased his mobility, and to encourage him out of old patterns. Alice recognized that Owen was more able to share activities with her and their son, which fulfilled her with joy. Prior to surgery, Owen had also undiagnosed sleep apnoea and depression, on which she did a lot of research, feeling that she could have provided more emotional support. Together with the issue of hiding when eating right after surgery, she regrets these shortcomings, which she relates to not having known enough about these problems. Alice considers that as a partner who doesn’t have the same problems, she sometimes might have worried too much, that it “took her a while to get on board” and that she was not always free of self-doubts. However, thanks to her listening attitude, she feels that she has learned to better understand and respond to Owen’s needs. Since her working hours changed (“she increased her hours”), she has less energy to structure meals and observes that her weight increased. Finally, Alice, who had herself counselling and therapy in the past, tells the interviewer that with food or other substances, things are laid bare once the person cannot compensate anymore, and that mental health support should then be offered and provided.

A1: I could see he was struggling with his health; I could see there were issues, and nothing seemed to work. And that definitively created a bit of a divide, a rift. A2: . . . but then we both did a lot of research and seeing him put the effort in and recognize that, you know, he was watching video of people having gone through it, sharing their story, sharing their struggle, and I think he felt understood. A3: I took pictures and little videos, I made him a little, kind of sounds cheesy, like a video montage thing of like, look at what you have done. And that was, you know, him sitting on a slide at the park, you know stuff that is limiting when you are a certain size, and climbing trees, so all that lovely stuff that he could do with us, because previously, I would just take the boys out to the park or the woods and he would be at home. A4: But I strongly believe that the root to a lot of you know, this, the issues with whatever substances, but in this case food, but there’s reasons behind it, and when you lose all that weight, and you can’t fulfil that comfort through food anymore, you kind of there’re other things, other factors that are laid bare.

The relational dynamics of the couple is characterized by individual changes and an evolution as a couple after surgery. While Owen acknowledges his initial denial regarding weight issues, thanks to Alice’s attitude confronting the weight problem, he was able to hear her “voice of reason” and work on himself. Alice appears psychologically minded, nuanced, supportive and creative and able to acknowledge some of her shortcomings, doubts and worries during the time she needed to adjust to the new situation. Both thus perceive intrapsychic difficulties and feel no need to avoid them by means of interpersonal defences, neither in the past (Owen’s initial denial is not an interpersonal defence and was not shared by Alice) nor in the present (a culture of honest communication prevails). Each partner had the right to experience the journey individually (Owen looking at patients witnessing, Alice eating alone right after surgery), and the couple grew closer. Their initial difference in experiencing weight issues decreased and the shared experiences increased. The changes following surgery was neither a threat for one of the partners nor for the couple. On the contrary: co-evolution and growth has taken place, benefitting each of the partners, the couple, and the family. Unlike the other couples described in this case series, this couple is not burdened by a past collusive relationship and therefore not threatened by change. One might judge that Owen remains more passive than Alice but accepting help when needed is not just passivity, it is an active engagement in an evolving process.

## Discussion

The present study used a psychodynamic and system approach to explore the constructs of sabotage, feeder behaviour and collusion, based on a case study approach. We will discuss these key concepts together with the notion of “co-evolution,” which appeared in the interviews considering the literature and our analytic perspective taken.

Sabotage can certainly be motivated by envy, guilt, suspicion of infidelity, fear of losing the partner or tensions over dietary restrictions. However, as observed by Ogden and Quirke-McFarlane (2023), these motivations are the consequences of a certain type of relationship and emerge in the context of a pre-existing relational dynamic. A close look at the former relationship may thus be necessary to understand how sabotage emerges in couples. Losing weight requires renegotiation and restructuring of one’s identity, which makes the reactions of partners inevitable; renegotiation is a difficult endeavour; even when partners’ feedback is positive; it may, for example, be interpreted as an indication that the former “self” was not truly accepted (Sarlio-Lahteenkorva, 1998). Especially in fragile relationships, and people living with obesity are for example more likely to experience conflict in marriage (Rand et al., 1982), obesity can stabilize the relationship with a partner (Neill et al., 1978) and weight regain can thus represent the return to a “save” normality. In other words: the “collaborating” victim of sabotage may also benefit, not physically, but emotionally. Moreover, collusion can be considered as a mutual exchange, with the saboteur, for example, benefiting from oral fulfilment by proxy (vicarious gratification), the victim benefiting from avoidance of an unresolved issue, and both benefiting from the re-establishment of a relational homeostasis.

This desire to re-establish the relational stability (Kluever Romo and Dailey, 2014) may be especially prevalent in couples with problematic attachment styles reacting with anxiety to threats of uncertainty. Indeed, researchers (Kiesewetter et al., 2010) found in a sample involving more than a hundred people living with obesity, that only about half of them (54%) showed a secure attachment style. One-fourth (25%) showed an insecure-avoidant attachment style, characterized by denied affect-burden, lack of intimacy and support in the past, a strive for independence, and trivialization of separation. About a fifth (21%) showed an insecure-ambivalent attachment style, characterized by overflooding affects, unprocessed drastic separations and massive dependency needs. Such attachment difficulties are associated with low self-esteem (Lee and Attachment, 2009), as illustrated by our cases.

Feeder behaviour may be motivated by the same anxieties as sabotage: the threat of a modification of the collusive relationship. In contrast to sabotage, feeding behaviour seems to be more active, which may be due to a personality trait of the feeding person or reflect the intensity of the collusive loss. In the couple illustrating feeding in this case series, the husband not only “lost his wife” as in the case of sabotage but his “mission” and relational identity of the past. This loss might be more important than the loss related to a modification of the partner’s, attitudes, and behaviours.

In couples, collusion may be at the very origin of the relationship (match) (Stiefel et al., 2024). Collusions can be intensified by triggers such as words, gestures, attitudes, acts, emotions, or specific situations with symbolic significance (Stiefel et al., 2023). The bodily changes of patients who undergo bariatric surgery may function as triggers, which intensify, reverse, or break collusions; sabotage and feeding or new relational interactions unrelated to weight management may be the consequence.

Collusion has already been observed in the clinics of obesity but not in the context of sabotage and feeding. Atkinson and McNamara (2017) observed that clinicians and patients collusively avoided to address the issue of obesity in consultations, which aimed to raise awareness about risk factors for pregnancy. Here, the unresolved issue at stake might be related to hurting and being hurt leading to shameful collusive avoidance. Also, in post-bariatric consultations, Natvik et al. observed that patients and clinicians collusively agreed to avoid bringing up difficult emotions related to the past (Natvik et al., 2023). Without calling it collusion, several authors describe persons who are “going along” with patients’ behaviour, which are not in line with their weight loss goals (Butterfield and Lewis, 2002; Kayman et al., 1990; Metzgar et al., 2015). Such behaviours might be considered as a milder form of sabotage. “Going along” may be motivated by separation-anxiety (fear of conflict) and considered collusive when partners share the same unresolved issue.

Finally, co-evolution, illustrated by the last interview is the most adequate response of couples facing a changed bio-psycho-social reality. Co-evolution, comprehensively described by Willi (37) who calls it the art of common growth, can be a goal of psychotherapeutic interventions, especially in situations of collusive negative social support.

## Clinical and scientific implications

Coming back to the title of our manuscript “And the winner is?,” we consider that our results call for an ecological perspective on obesity, taking into account biological, psychological, social, and cultural dimensions. An ecological perspective conceives that there are always winners and losers in cases of collusive weight gain after bariatric surgery. Sometimes the win is situated on a intrapsychic level and the body is losing; sometimes the winners are the patients, and their partners lose or vice versa; and sometimes both partners are apparent winners but also losers, since they miss an opportunity to co-evolve and learn from each other instead of remaining in roles fixed by their past. This perspective is inserted in a long tradition of thought, which not only encompasses views on physiological homeostasis but also Freudian metapsychology and more recent scholars’ theories of brain functioning such as Domasio (for a review, see (Arminjon et al., 2010)). An ecological understanding requires a clinical approach, which includes a dyadic perspective in weight management, especially for couples at risk for sabotage, feeding behaviour or other collusive developments. The newly developed questionnaires to identify negative social support (Ogden et al., 2020) might be of help to target such interventions.

Finally, from a research perspective, we consider that qualitative methodologies are appropriate to investigate the more subtle phenomena of intrapsychic phenomena such as ambivalence (38) or interpersonal defensive manoeuvres such as collusion.

## Limitations of the study

There are several limitations of the study. A case study analysis can at best explore a phenomenon and pave the way for further investigations. Therefore, our observations must be considered as hypotheses. Second, the interview guide was not developed to identify collusive relationships. Third, interviews narrowly focused on support without addressing participants’ development, life events and other aspects of their life. Fourth, the arguments might be considered as somehow circular, since we select data which confirm the perspective taken on interactional processes operating in these couples. We do agree that we did not select the quotes which speak in favour of positive support or of non-collusive relational elements in the couple relationships. However, we would like to remind that in a psychodynamic view, psychic processes are considered as being the results of different forces, and there is no linear and causal relationship between a force and a resulting behaviour. The identification of collusive elements is thus not considered as the sole source of negative social support, but as a way of “reading” and understanding some aspects of the interpersonal dynamics of sabotage and feeder behaviour. Finally, there is no other way of “prove” the existence of collusion than arguing for the interpretations one makes about a relational dynamic; we hope that our arguments appear plausible to the readers.

## Conclusions

Our case study analysis illustrates that an ecological approach to negative social support, considering exchanges on different levels—here regarding the intrapsychic and interpersonal dimensions—seems necessary to apprehend sabotage and feeding behaviour. In line with Ogden and Quirke-McFarlane’s model (2023), negative social support can be understood as a relational dynamic, which attempts to reestablish a level of homeostasis within the couple. In contrast, however, we suggest that rather being intentional, sabotage and feeder behaviour are better conceptualised as consequences of collusive relationships.

A systemic and a psychodynamic perspective allows, from our point of view, to discover mechanisms at work in negative social support; collusion - as a concept unifying intrapsychic and interpersonal perspectives - could be an interesting framework to guide future interventions and research.

Since psychological hypotheses and ways of understanding psychic phenomena remain difficult to “prove,” the evaluation of therapeutic interventions targeting negative social support, based on a combined psychodynamic and systemic approach, would be a way to validate our assumptions. Moreover, it would also help people living with obesity and their partners, who experience negative social support, instead of judging these phenomena on moral grounds. As seen in our study, both the person subjected to negative social support and the person who practice it, are suffering and deserve our attention.

## Supplemental Material

sj-docx-1-hea-10.1177_13634593251319928 – Supplemental material for Sabotage, feeding and collusion after bariatric surgery. And the winner is . . .? A psychodynamic and systemic perspective on sabotage and feeding after bariatric surgery by means of a case series analysis Supplemental material, sj-docx-1-hea-10.1177_13634593251319928 for Sabotage, feeding and collusion after bariatric surgery. And the winner is . . .? A psychodynamic and systemic perspective on sabotage and feeding after bariatric surgery by means of a case series analysis by Friedrich Stiefel, Laurent Michaud, Céline Bourquin-Sachse, Sophia Quirke-McFarlane and Jane Ogden in Health
