# Supplementary material for: Sabotage, feeding and collusion after bariatric surgery. And the winner is . . .? A psychodynamic and systemic perspective on sabotage and feeding after bariatric surgery by means of a case series analysis
Source: Health (London). 2025 Feb 27;30(1):59–80. doi: 10.1177/13634593251319928 (PMC12741170; doi:10.1177/13634593251319928)
Supplement: sj-docx-1-hea-10.1177_13634593251319928 – Supplemental material for Sabotage, feeding and collusion after bariatric surgery. And the winner is . . .? A psychodynamic and systemic perspective on sabotage and feeding after bariatric surgery by means of a case series analysis [file sj-docx-1-hea-10.1177_13634593251319928.docx]

**Table 1**

*Semi-Structured Interview Guide for patients*

| Procedure | Summary of Procedure |
| --- | --- |
| Introduction | “Thank you for your participation in this discussion. I’m interested in understanding how your current or previous partner has supported you throughout your bariatric surgery journey. Some people have said that their partner has been a big support throughout this journey, whilst others have said that their partners have been less supportive throughout.” |
| Partner social support definition | “Throughout the interview, we will discuss the concept of partner social support. This concept may mean different things to different people. For some, the support they receive from their partner might be solely emotional, whilst for others it may be more practical such as a partner taking their significant other to a hospital appointment. In this interview, please feel free to speak about partner social support in the terms in which you understand it.” |
| Exploration of partner social support received pre-operatively | Thinking back to before your bariatric surgery, can you please tell me the ways in which you feel your partner supported you when you were trying to lose weight? |
| Exploration of partner social support received during the decision-making process | Now thinking about when you were considering bariatric surgery, in what ways, if any, was your partner supportive at this time? |
| Exploration of partner social support received post-operatively | Following your bariatric surgery, in what ways, if any, has your partner been supportive?  In the time since your bariatric surgery, is there anything that your partner has said or done that has been especially *helpful* to you in adjusting to your bariatric surgery? If so, what was that?  In the time since your bariatric surgery, is there anything that your partner has said or done that has been especially *unhelpful* to you in adjusting to your bariatric surgery? If so, what was that? |
| Finishing up | Is there anything else relating to partner social support that you would like to discuss in this interview? |

*Partner semi-structured interview guide*

| Procedure | Summary of Procedure |
| --- | --- |
| Introduction | Thank you for your participation in this discussion. I’m interested in understanding your experience of and feelings about your current or previous partner’s weight loss surgery journey.” |
| Support from partner before the surgery, during the surgery decision making process, and after the surgery | - Can you talk me through why your partner decided to have weight loss surgery and how you felt about this?   - What were the good things and not so good things? - Do you feel like the surgery has changed your relationship in anyway? How have you felt about this?   - Prompts: Home life; Household roles; Caregiving responsibilities; Communication; Sexual intimacy; Eating habits   - Good and not so good things - Sometimes people have said whilst surgery has been a good thing, it isn’t always easy to manage. How have you found it?   - What changes have/had been the most difficult to adjust to? Please explain.   - What changes have/had been easiest to adjust to? Please explain. - Looking back, do you think your partner would say you have been supportive throughout their weight loss surgery journey?   - In what ways? - Have they ever said you were less than supportive? |
| Finishing up | Is there anything I did not ask you related to your relationship after weight loss surgery that you think is relevant to this study? |
